# Effect of acupuncture treatment on cognitive impairment after traumatic brain injury in adults: A protocol for systematic review

**DOI:** 10.1097/MD.0000000000028451

**Published:** 2021-12-23

**Authors:** Na Li, Ruihui Wang, Xia Ai, Xinrong Guo, Juan Liu, Lei Sun, Rongchao Zhang

**Affiliations:** a School of Acupuncture-Tuina, Chengdu University of Traditional Chinese Medicine, Chengdu, China; b School of Acupuncture-Tuina, Shaanxi University of Traditional Chinese Medicine, Xi’an, Shaanxi, China.

**Keywords:** acupuncture, cognitive impairment, protocol, randomized controlled trials, systematic review, traumatic brain injury

## Abstract

**Background:**

Acupuncture has been widely used to treat cognitive impairment after traumatic brain injury (TBI). But its efficiency has not been scientifically and methodically evaluated. The objective of this study is to evaluate the efficiency and safety of the acupuncture treatment for cognitive impairment after TBI in adults.

**Methods:**

This protocol of systematic review will be conducted in accordance with the Preferred Reporting Items for Systematic Review and Meta-analysis Protocols. We will conduct the literature searching in the following electronic databases: the Cochrane Library, MEDLINE, EMBASE, Web of Science, Springer, the Chinese Science Citation Database (CSCD), China National Knowledge Infrastructure (CNKI), the Chinese Biomedical Literature Database (CBM), Wanfang, and the Chinese Scientific Journal Database (VIP). The time limit for retrieving studies is from establishment to November 2021 for each database. All published randomized controlled trials related to this review will be included. Review Manager (V.5.3.5) will be implemented for the assessment of bias risk and data analyses. The selection of the studies, data abstraction, and validations will be performed independently by 2 researchers.

**Results:**

This review will assess the clinical efficacy and safety, as well as the acupoints characteristics of acupuncture on CI of TBI in adults.

**Conclusion:**

This review will summarize the current evidence of acupuncture on CI of TBI outcomes and provide guidance for clinicians and patients to select acupuncture for CI of TBI in adults.

**Trail registration number:**

This protocol of systematic review has been registered on INPLASY website (No. INPLASY2021110113).

## 1 Introduction

### 1.1 Description of the condition

Traumatic brain injury (TBI) is among the significant causes of morbidity and mortality in the present world.^[1,2]^ About 1.7 million individuals yearly in the United States and causing 1.5 million hospitalizations in the European Union yearly.^[3,4]^ From 2006 to 2014, there was a 53% increase in the number of TBI-related emergency department visits, hospitalizations, and deaths. Approximately 155 people die each day in the US from injuries that include a TBI.^[5]^

Cognitive symptoms are an especially common and important area of concern for TBI survivors, with a majority of individuals with moderate-to-severe TBI having persistent and marked cognitive impairment (IC) at 1 to 5 years post-injury.^[6,7]^ Cognitive symptoms include deficits in attention, executive functioning, memory (encoding and retrieval), language, visuoperceptual functioning, and psychomotor functioning abilities.^[8–10]^ Cognitive deficits can significantly impair activities of daily living (ADL), employment, social relationships, recreation, and active participation in the community.^[11]^ Cognitive rehabilitation is a confluence of therapeutic activities based on brain–behavior relationships. Cognitive rehabilitation includes methods such as remediation, compensation, and holistic or multimodal programs.^[12,13]^

Acupuncture therapy is an external therapy used in traditional Chinese medicine that has been using as an alternative nonpharmacological therapy that involves the insertion of needles into acupuncture points in the skin as to correct imbalances of the flow of Qi through meridians.^[14]^ Nowadays, acupuncture has been widely used clinically by doctors of traditional Chinese Medicine, neuropsychiatric disorder to treat IC of traumatic brain injury with satisfied efficacy.^[15–18]^ However, no relevant review or protocol has been published to date. Therefore, it is necessary to conduct evidence-based review to evaluate the efficacy and safety of acupuncture for FC in adults. It is urgently needed to accomplish this review.

## 2 Methods

The protocol for this review has been registered in the International Platform of Registered Systematic Review and Meta-analysis Protocols (INPLASY) (registration number: INPLASY2021110113) on November 30, 2021. Available online: https://inplasy.com/?s=INPLASY2021110113. The protocol will be strictly developed under the guidelines of the Preferred Reporting Items for Systematic Reviews and Meta-Analyses protocols,^[19]^ and Cochrane Handbook for Systematic Reviews of Interventions.^[20]^

### 2.1 Selection criteria

#### 2.1.1 Types of studies

As the randomized controlled trials (RCTs) are reliable and feasible, RCTs will be included only. After the research, published clinical trials that reported the efficacy and safety on acupuncture for IC of TBI will be included. Literature of non-RCTs, case reports, animal research, meta-analyses, reviews, and retrospective studies will be excluded.

#### 2.1.2 Types of patients

Patients aged ≥18 years and diagnosed with IC of TBI according to Montreal cognitive assessment scale (MoCA) <26 will be included, without limits on age, gender, nationality, race, and medical units.

#### 2.1.3 Types of interventions and comparisons

We will include the studies using acupuncture as the sole intervention in the experimental group, while we have no restrictions on intervention in the control group. Studies involving acupuncture combined with other therapies will be included if the other therapies are used equally in both the experimental and control groups.

#### 2.1.4 Types of outcomes

The main outcomes of this review include Montreal cognitive assessment scale (MoCA). Additional outcomes of this review include Mini-mental State Examination (MMSE), and Barthel index (BI).

### 2.2 Search methods for the identification of studies

#### 2.2.1 Electronic search strategy

The electronic databases of the Cochrane Library, MEDLINE, EMBASE, Web of Science, Springer, the Chinese Science Citation Database (CSCD), China National Knowledge Infrastructure (CNKI), the Chinese Biomedical Literature Database (CBM), Wanfang, and the Chinese Scientific Journal Database (VIP) will be searched from the establishment to July 1, 2020. All published RCTs on this subject will be included. Exemplary search strategy of MEDLINE is listed in Table 1, and terms are conformed to the medical subject heading. According to the different retrieval modes, keywords may combine with free words and comprehensive search will be performed.

**Table 1:** Search strategy used in MEDLINE database.

#1 Title/Abstract: Brain Injury, traumatic
#2 Title/Abstract: Traumatic brain injuries
#3 Title/Abstract: Traumatic brain injury
#4 Title/Abstract: TBI
#5 Title/Abstract: Brain trauma
#6 Title/Abstract: Brain traumas
#7 Title/Abstract: Traumas, brain
#8 Title/Abstract: Trauma, brain
#9 Title/Abstract: Encephalopathy, traumatic
#10 Title/Abstract: Traumatic encephalopathy
#11 Title/Abstract: Head injury
#12 Title/Abstract: Closed head injury
#13 Title/Abstract: Closed head injuries
#14 Title/Abstract: Head injury, closed
#15 Title/Abstract: Head trauma
#16 Title/Abstract: Head injury, nonpenetrating
#17 Title/Abstract: Head injuries, nonpenetrating
#18 Title/Abstract: Blunt head injury
#19 Title/Abstract: Blunt head injuries
#20 Title/Abstract: Head injuries, blunt
#21 Title/Abstract: Prefrontal cortex damage
#22 OR 1-21
#23 Title/Abstract: Cognitive Dysfunctions
#24 Title/Abstract: Dysfunction, Cognitive
#25 Title/Abstract: Dysfunctions, Cognitive
#26 Title/Abstract: Cognitive Impairments
#27 Title/Abstract: Cognitive Impairment
#28 Title/Abstract: Impairment, Cognitive
#29 Title/Abstract: Impairments, Cognitive
#30 Title/Abstract: Mild Cognitive Impairment
#31 Title/Abstract: Cognitive Impairment, Mild
#32 Title/Abstract: Cognitive Impairments, Mild
#33 Title/Abstract: Impairment, Mild Cognitive
#34 Title/Abstract: Impairments, Mild Cognitive
#35 Title/Abstract: Mild Cognitive Impairments
#36 Title/Abstract: Mild Neurocognitive Disorder
#37 Title/Abstract: Disorder, Mild Neurocognitive
#38 Title/Abstract: Disorders, Mild Neurocognitive
#39 Title/Abstract: Mild Neurocognitive Disorders
#40 Title/Abstract: Neurocognitive Disorder, Mild
#41 Title/Abstract: Neurocognitive Disorders, Mild
#42 Title/Abstract: Cognitive Decline
#43 Title/Abstract: Cognitive Declines
#44 Title/Abstract: Decline, Cognitive
#45 Title/Abstract: Declines, Cognitive
#46 Title/Abstract: Mental Deterioration
#47 Title/Abstract: Deterioration, Mental
#48 Title/Abstract: Deteriorations, Mental
#49 Title/Abstract: Mental Deteriorations
#50 OR 23-49
#51 Title/Abstract:Acupuncture therapy
#52 Title/Abstract:Acupuncture therapy
#53 Title/Abstract: Pharmacoacupuncture treatment
#54 Title/Abstract: Pharmacoacupuncture therapy
#55 Title/Abstract: Acupuncture
#56 Title/Abstract: Acupoints
#57 Title/Abstract: Acupunct
#58 Title/Abstract: Manual acupuncture
#59 Title/Abstract: Body acupuncture
#60 Title/Abstract: Scalp acupuncture
#61 Title/Abstract: Auricular acupuncture
#62 Title/Abstract: Auriculotherapies
#63 Title/Abstract: Electroacupuncture
#64 Title/Abstract:Fire needling
#65 Title/Abstract: Warm needling
#66 Title/Abstract: Elongated needle
#67 Title/Abstract: Intradermal needling
#68 Title/Abstract: Dermal needle
#69 Title/Abstract: Plum blossom needle
#70 OR 51-69
#71 Title/Abstract: randomized controlled trial
#72 Title/Abstract: controlled clinical trial
#73 Title/Abstract: randomized
#74 Title/Abstract: randomly
#75 Title/Abstract: RCT
#76 Title/Abstract: trial
#77 OR 71-76
#78 Title/Abstract: ≥18 years of age
#79 Human
#80 #22 AND #50 AND #70 AND #77AND#78 AND#79

Relevant keywords were used to create search strategies, as listed in Table 1. In the selection process, only research conducted in humans will be included in further review.

The MEDLINE search strategy in Table 1 will be adapted for other databases.

### 2.3 Data extraction, quality, and validation

#### 2.3.1 Study inclusion

**Figure 1:**
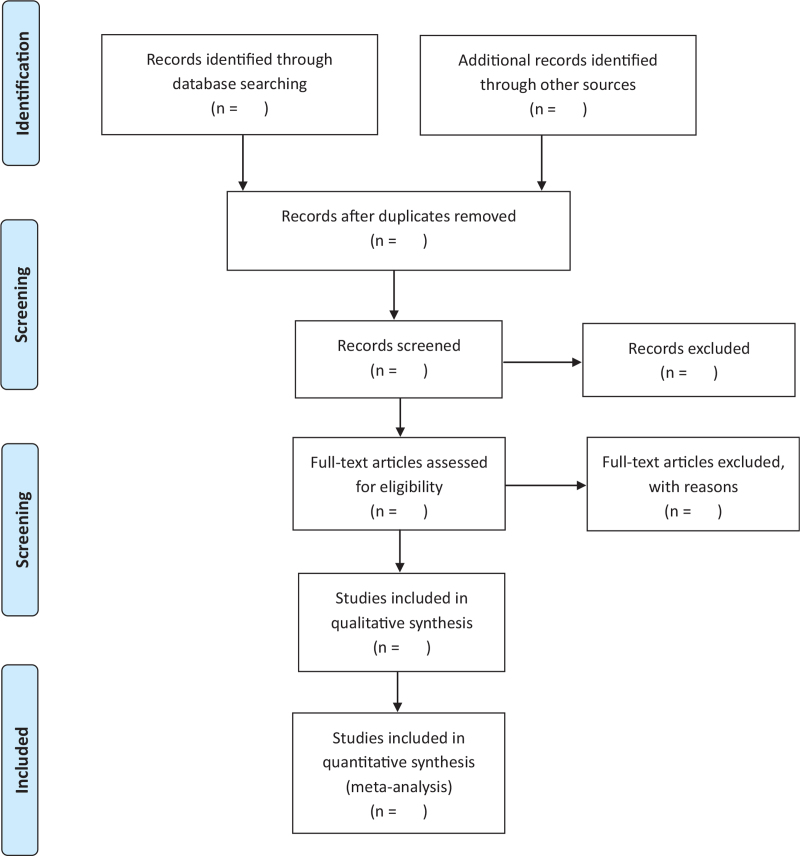
PRISMA flow diagram of study process. PRISMA = preferred reporting items for systematic reviews and meta-analysis.

Two reviewers (NL and XA), who will be told the aim and process of the system review, will select the trials and studies independently according to the criteria for inclusion by reading the titles and abstracts; if necessary, the full text will be read for further assessment. The discrepancies in the process will be discussed and solved using RHW. Details of the research choices are shown and exclusive studies will be listed and explained. The process of study selection will be presented in Preferred Reporting Items for Systematic Reviews and Meta-Analyses flow diagram (Fig. 1). To ensure consistency, we will perform calibration exercises on methodological steps of the review process before assessment.

#### 2.3.2 Data extraction and management

An electronic form will be established via Excel to extract substantial contents, and then filled by 2 reviewers independently. Information was extracted from the qualified articles by using a standardized data extraction form as follows: authors’ name, country, year of publication, study design, gender, and age of patients, intervention, sample size, outcomes, adverse events, and follow-up. Disagreements will be solved by group discussion or consult seniors. However, if we fail to reach a consensus, the authors of trials will be contacted for further details and verification.

#### 2.3.3 Managing missing data

If any data information is not sufficient in the included trials, we will try to contact the first or corresponding author by phone or email, requesting adequate information and details of the studies included to retrieve missing or insufficient trial data. However, if the author is not available or sufficient information cannot be obtained, we will have a group discussion and analysis based on the current information. Meanwhile, the potential impact of missing data will be taken into account, and a relative discussion will be presented in the result section.

#### 2.3.4 Assessment of bias risk and quality of the included studies

Two independent authors assess the risk of bias of each included article according to the Cochrane Handbook for Systematic Reviews of Interventions. The methodological quality will be evaluated from the following domains: random sequence generation, allocation concealment, blinding of participants, and therapists, blinding of outcome assessment, incomplete outcome data, selective reporting, and other biases. The judgements on these items will be categorized as “high risk of bias”, “low risk of bias”, or “unclear risk of bias”. Discrepancies will be resolved by negotiation or by consulting other reviewers.

#### 2.3.5 Measures of treatment effects

Dichotomous data and continuous variables are included in the outcomes of interest, and we will use the risk ratio (RR) to express dichotomous data and use mean difference (MD) to assess differences in the continuous outcomes between the groups. Although different methods for the measurement of outcomes are used in different trials, we will choose standardized mean difference (SMD) if they have the same outcomes. The corresponding 95% confidence interval (CI) for each parameter will be calculated between the electroacupuncture treated group and the control group. We will choose a descriptive review if quantitative synthesis is not appropriate.

#### 2.3.6 Assessment of heterogeneity

The research will be performed with RevMan 5.3.5 software. *P* < .5 will be defined as statistically significant between studies. The heterogeneity of studies will be evaluated by *I*^2^ statistic. The following criteria will be used: *I*^2^ < 50% will be deemed as low heterogeneity; *I*^2^ between 50% and 75% will be considered as moderate heterogeneity; *I*^2^ > 75% will be considered as high heterogeneity.

#### 2.3.7 Subgroup analysis

When heterogeneity is high, we will perform a subgroup analysis based on different controls, intervention time, treatment frequency, follow-up duration, and outcome measurements. We will also tabulate the adverse reactions and then perform an evaluation.

#### 2.3.8 Sensitivity analysis

If possible, a sensitivity analysis will be performed to verify the robustness of the review conclusions. The impact of methodological quality, sample size, and missing data will be assessed. In addition, the analysis will be repeated after the exclusion of low methodological quality studies.

## 3 Discussion

IC of TBI is one source of distress. This disease has brought physical, psychological, and economic burdens to many people in many countries and has brought great suffering. It has aroused more and more concern all over the world. As an important part of traditional Chinese medicine, Acupuncture has been widely used in clinical practice, especially in the treatment of nervous system diseases, for its various therapeutic techniques and remarkable curative effects. Now, Acupuncture has become more and more widely used in IC of TBI because it is a relatively straightforward, safe, and cheap therapy, compared with other conventional therapies. But at the present, there is no systematic evaluation report on its therapeutic effectiveness and safety for the treatment of IC of TBI.

The protocol of this systematic review study aims to assess the efficacy, safety, and cost benefits of acupuncture in the treatment of IC of TBI in adults. Meanwhile, we have tried our best to search and found that no relevant systematic review and meta-analysis concerning this topic has been reported in the last 5 years, and we will integrate the latest and most comprehensive clinical evidence in this field, hoping to offer a greater variety of treatment options for patients with IC of TBI and inspire more peer experts and doctors to carry out as many relevant studies as possible in the future.

## Author contributions

**Conceptualization:** Na Li, Ruihui Wang.

**Data curation:** Na Li, Xia Ai, Xinrong Guo, Juan Liu, Dong Wang, Lei Sun, Rongchao Zhang.

**Investigation:** Xinrong Guo.

**Methodology:** Na Li, Xia Ai.

**Validation:** Ruihui Wang.

**Visualization:** Na Li.

**Writing – original draft:** Na Li, Xia Ai.

**Writing – review & editing:** Ruihui Wang, Lei Sun.
